# Beta-Blocker Use and Self-Reported Sleep Changes Among Medical Students in Jordan: A Cross-Sectional Study

**DOI:** 10.3390/healthcare14152257

**Published:** 2026-07-24

**Authors:** Mohammad Alzoubi, Wisam Al Safadi, Selina Sorour, Rami Ammori, Abdulrahman Ahmad, Yara Dawud, Fadia Shalash, Mona Mohammad, Maha T. Mohammad

**Affiliations:** 1 Department of General Surgery, School of Medicine, The University of Jordan, Amman 11942, Jordan; moh_alzoubi@ju.edu.jo; 2 School of Medicine, The University of Jordan, Amman 11942, Jordan; wsa2210039@ju.edu.jo (W.A.S.); syl0215140@ju.edu.jo (S.S.); ram2210027@ju.edu.jo (R.A.); abd2210202@ju.edu.jo (A.A.); yar0204708@ju.edu.jo (Y.D.); fad0215454@ju.edu.jo (F.S.); 3 Department of Surgery/Ophthalmology, King Hussein Cancer Center, Amman 11941, Jordan; 4 Department of Physiotherapy, The University of Jordan, Amman 11942, Jordan

**Keywords:** beta-blockers, sleep disturbances, sleep quality, non-prescribed drug use, medical students

## Abstract

**Highlights:**

**What are the main findings?**

- Beta-blocker use among Jordanian medical students was low (4.9%); however, it was predominantly non-prescribed use and mainly driven by performance anxiety.
- Smoking status was significantly associated with beta-blocker use, and male students had lower odds of beta-blocker use compared with females (OR = 0.4). Users also reported a high burden of sleep disturbances, including nocturnal awakenings, nightmares, and morning fatigue.

**What are the implications of the main findings?**

- Medical schools and health services should raise awareness about the potential sleep-related adverse effects of non-prescribed beta-blocker use for performance anxiety, particularly among medical students.
- Educational and counselling efforts should address the unsupervised widespread use of beta-blockers and promote safer management of performance-related anxiety.

**Abstract:**

**Introduction**: Sleep is essential for cognitive performance and psychological well-being. Medical students are particularly vulnerable to sleep disturbances due to academic stress and irregular schedules. Beta-blockers are increasingly used to alleviate anxiety and performance stress, with lipophilic agents such as propranolol affecting sleep. Evidence on the association of beta-blocker use with sleep disturbances among medical students in Jordan is limited. **Objective**: The aim of this study was to describe self-reported sleep characteristics among medical students in Jordan and report patterns related to beta-blocker use. **Methods**: A quantitative cross-sectional study was conducted among 697 undergraduate students from six public medical schools in Jordan over a one-month period. Data were collected using an anonymous online questionnaire on beta-blocker use and sleep characteristics. Associations were analyzed using chi-square tests, with a *p* < 0.05 considered significant. **Results**: Of 697 students, 23 (3.3%) were current and 11 (1.6%) were previous users of beta-blockers; 663 (95.1%) had never used them. Beta-blocker use was significantly associated with smoking status (*p* = 0.013) but not with academic level or caffeine consumption. In multivariable analysis, males had lower odds (OR = 0.4) of beta-blocker use compared with females. More than half of current users (56.5%) reported non-prescribed use, with propranolol (78.3%) being the most commonly used beta-blocker. Sleep-related disturbances were frequently reported among current users, including nocturnal awakenings (73.9%), nightmares or disturbing dreams (69.6%), morning fatigue (65.2%), difficulty waking (52.2%), and sleep-onset insomnia (39.1%), with about one-third reporting a decline in sleep quality. **Conclusions**: Beta-blocker use among medical students in Jordan is mostly non-prescribed and primarily for anxiety or exam stress. Users frequently reported sleep disturbances, highlighting the need for medical guidance to prevent adverse effects. These findings can inform local guidelines and raise awareness about the risks of unsupervised beta-blocker use among medical students, supporting safer practices and better overall well-being.

## 1. Introduction

Medical students face high levels of stress and performance-related anxiety due to heavy academic workloads, frequent examinations, and competitive environments [1,2,3]. To cope with these challenges, some students seek pharmacological treatments, including the use of beta-blockers, especially for the management of situational anxiety during exams and presentations [4,5,6].

Sleep plays a fundamental role in maintaining both physical and mental health, as well as overall quality of life [7]. It is essential for cognitive and physiological processes such as concentration, memory, learning, mood regulation, and daily performance [8]. Disruptions in sleep have been shown to negatively affect cognitive performance, psychological well-being, and overall functioning [7,9].

Medical students represent a population particularly vulnerable to sleep disturbances due to high academic demands, irregular schedules, and high levels of stress. Several surveys have reported that medical students frequently experience insufficient sleep, particularly during examination periods, often averaging 5–6 h per night, with a substantial proportion sleeping four hours or less [10]. Poor sleep in this population has been consistently associated with impaired concentration, reduced academic performance, and adverse mental health outcomes [9,10,11,12].

Concurrently, beta-blockers are commonly used medications that act by blocking β-adrenergic receptors and are prescribed for cardiovascular conditions, as well as for migraine prophylaxis and situational anxiety [13,14]. Among students, their use has increasingly extended to the management of performance-related anxiety. Studies have reported that approximately 5–12% of medical students use beta-blockers often without medical supervision and frequently based on peer recommendations [10,15]. Regional findings reflect similar patterns, with a notable proportion of students reporting non-prescribed use [6].

Beta-blockers may affect sleep through their central nervous system effects, which vary according to their ability to cross the blood–brain barrier. Lipophilic agents such as propranolol can penetrate the central nervous system and are associated with fatigue, somnolence, insomnia, and other sleep disturbances [14,16]. However, hydrophilic agents such as atenolol are less likely to produce these effects. Evidence regarding their association with sleep remains mixed. A systematic review and meta-analysis of randomized controlled trials reported a small, non-statistically significant increase in insomnia among beta-blocker users compared with placebo [14]. Moreover, data from the National Health and Nutrition Examination Survey (NHANES) (2011–2018) showed that beta-blocker users were more likely to report sleep-related complaints despite longer total sleep duration, suggesting poorer sleep quality [17].

However, beta-blocker use among medical students who are already predisposed to poor sleep makes it difficult to interpret how these medications relate to students’ self-reported sleep experiences in real-world settings [17,18]. Despite growing international and regional evidence, there is limited data describing patterns of beta-blocker use and associated self-reported sleep characteristics among medical students, particularly in Jordan.

Therefore, this study aims to describe beta-blocker use among medical students in Jordan, including patterns of use, perceived effectiveness, and adverse effects, and to characterize self-reported sleep patterns and characteristics among current users. Additionally, the study compares selected demographic characteristics between users and non-users. This contributes to a better understanding of how beta-blocker use relates to self-reported sleep characteristics in a population where both stress-related medication use and sleep disturbances are common.

## 2. Methods

### 2.1. Study Design

This is a quantitative cross-sectional study that was conducted in six public medical schools in Jordan (Hashemite University, Jordan University of Science and Technology, Mutah University, University of Jordan, Yarmouk University, and Al-Balqa Applied University). Data were collected using an anonymous, self-administered online questionnaire that was distributed to undergraduate medical students attending those universities. The study followed the Strengthening the Reporting of Observational Studies in Epidemiology (STROBE) guidelines.

### 2.2. Participant Selection and Sampling

The study included male and female undergraduate medical students from first to sixth year, enrolled in public medical schools in Jordan during the 2025–2026 academic year. Post-graduate students were excluded.

### 2.3. Sample Size and Sampling Method

Participants were selected using a convenience sampling method. The sample size was calculated using Cochran’s formula, with a 95% confidence level, 5% margin of error, and an estimated prevalence of beta-blocker use of 10% based on regional literature [12,13,14]. The study required a minimum of 139 participants. To account for non-response, the target number was increased to 276 students. A larger sample (*n* = 697) was included to improve precision and ensure adequate representation.

### 2.4. Questionnaire

A structured questionnaire was developed based on prior literature [6,19,20]. The questionnaire was checked for clarity and face validity by the research supervisor and was pilot-tested on a small group of students prior to distribution to ensure comprehensibility and clarity of wording. The questionnaire was administered in Arabic language, it included a section that collected sociodemographic characteristics; including age, gender, academic year, marital status, smoking status, and caffeine consumption, which was defined as self-reported intake of coffee, tea, and energy drinks; a section on beta-blocker use patterns which included indications, source of information, prescription status, type, duration, frequency, perceived effectiveness, and adverse effects; and a section on post-beta-blocker sleep characteristics, which were assessed descriptively using questions on sleep duration, sleep latency, nocturnal awakenings, sleep-onset insomnia, nightmares, daytime fatigue, difficulty waking up, morning sleepiness, and use of sleep aids. Awareness of long-term risks was assessed using a single binary (yes/no) self-reported question. The original questionnaire (in Arabic) and a translated version (in English) are provided in the Supplementary Material.

### 2.5. Data Collection

The survey was created using Google Forms (Google LLC, Mountain View, CA, USA) and distributed online to medical student groups via social media platforms, including WhatsApp and Facebook (Meta Platforms, Inc., Melano Park, CA, USA). Participation was voluntary and anonymous. The data collection was conducted over a one-month period. The online questionnaire required mandatory responses to all items; therefore, incomplete questionnaires were not submitted, and no participants were excluded due to missing data.

### 2.6. Statistical Analysis

An Excel spreadsheet was used to arrange and clean the data. Statistical analysis was performed using R software version 4.6.0 (R Foundation for Statistical Computing, Vienna, Austria) and figures were generated using the same software. Descriptive statistics were used to summarize demographic variables and patterns of beta-blocker use. Categorical variables were presented as frequencies and percentages. Associations between beta-blocker use and demographic, lifestyle, and related variables were assessed using Pearson’s Chi-square test.

Smoking status was originally recorded as three categories (non-smoker, smoker and ex-smoker), and the academic level was originally collected as six separate categories (first to sixth year). For descriptive analysis, they were retained in their original categories. For chi-square analysis and the multivariable logistic regression model, smoking was recorded as a binary variable, where current smokers and ex-smokers were combined into a single category (“ever smokers”), while non-smokers were used as the reference category (“never smokers”). Academic level was further collapsed into two categories: preclinical years (first to third year) and clinical years (fourth to sixth year). This categorization was used to ensure adequate expected cell counts and consistency across statistical models.

Multivariable logistic regression analysis was performed to evaluate factors associated with beta-blocker use while adjusting for sex and academic level. Variables included in the regression model were smoking status (ever smoker vs. never smoker), sex, and academic level (preclinical years vs. clinical years). Results were presented as odds ratios (OR) with 95% confidence intervals (95% CI) and corresponding *p*-values.

For descriptive analyses, proportions and corresponding 95% confidence intervals were calculated using the Wilson binomial method. For variables with more than two categories, confidence intervals were calculated separately for each category proportion.

Statistical significance was set at *p* < 0.05 for all analyses.

### 2.7. Ethical Considerations

An ethical approval was obtained from the Research Ethics Committee at Jordan University Hospital (approval number: 10/2025/30847). Participants were given sufficient information regarding the objectives of the study and the nature of their participation, in addition to their right to withdraw at any time without any consequences. Participation in the study was entirely voluntary, without any pressure from any party on the participants, and consent was obtained from all involved individuals. The identity of the participants was fully preserved, as all collected data was securely stored and used solely for research purposes.

## 3. Results

### 3.1. Sociodemographic Characteristics of the Study Population

**Figure 1:**
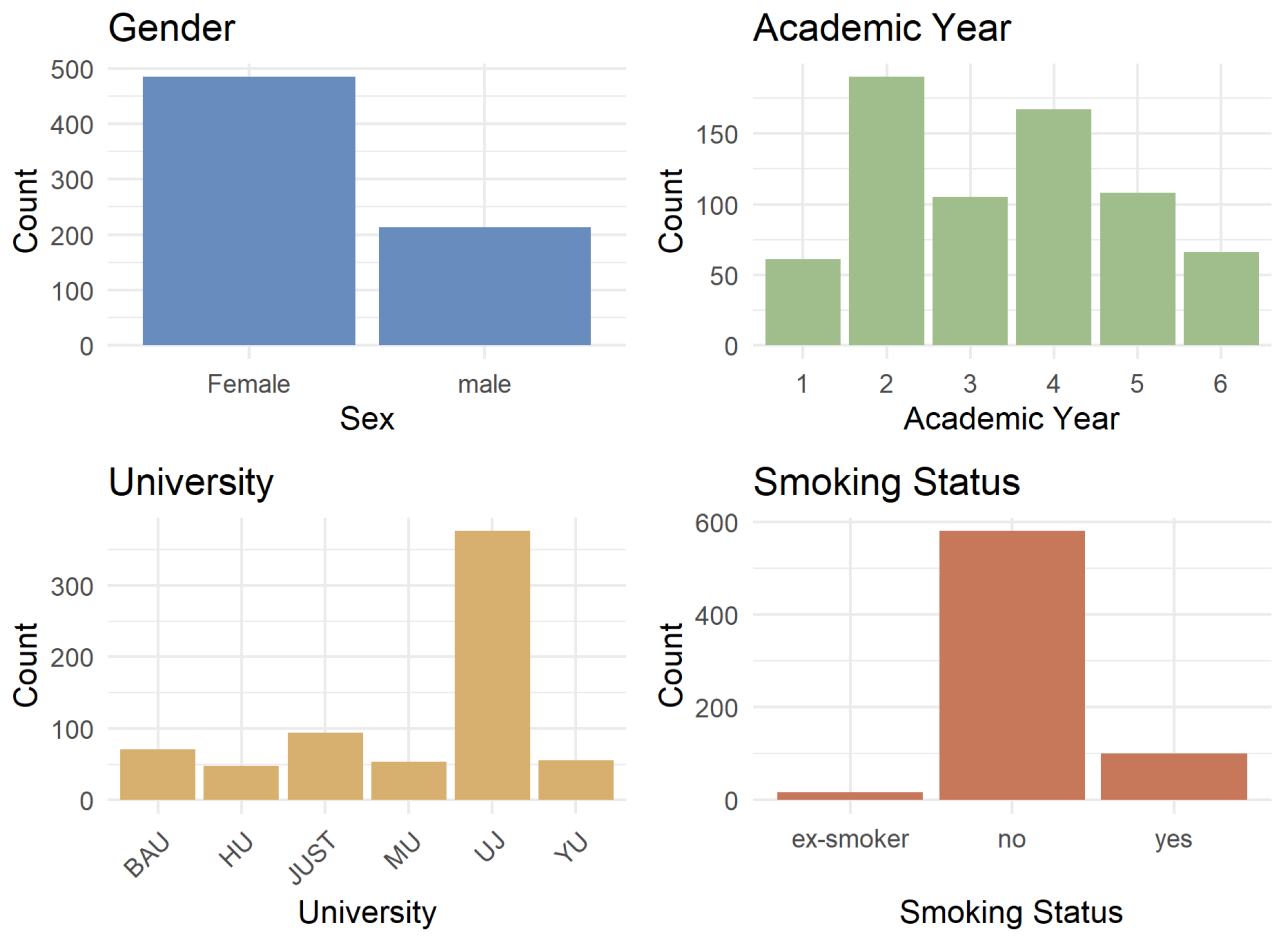
Demographic characteristics of the study sample. BAU = Al-Balqa Applied University; HU = Hashemite University; JUST = Jordan University of Science and Technology; MU = Mutah University; UJ = the University of Jordan; YU = Yarmouk University.

A total of 697 medical students were included in the study. The majority (67.4%) were aged 18–21 years, and females constituted 69.4% of the sample. Students from all academic years were represented, with the highest proportions in the second year (27.3%) and fourth year (24.0%). In addition, 53.9% of them were from the University of Jordan. Most participants were single (98.6%), non-smokers (83.2%), and reported caffeine consumption (85.8%). Table 1 and Figure 1 summarize participants’ demographics.

The majority of students in the study sample (*n* = 663, 95.1%) reported never using beta-blockers. A total of 34 students (4.9%) reported beta-blocker use, including 23 (3.3%) current beta-blocker users, and 11 (1.6%) past users (Table 1).

### 3.2. Association Between Beta-Blocker Use and Demographic Factors

Smoking status was significantly associated with beta-blocker use (χ^2^ = 6.201, *p* = 0.013), with a higher proportion of beta-blocker users among ever smokers (9.4%) than never smokers (4.0%). No significant association was found between beta-blocker use and gender, academic level or caffeine consumption (Table 2).

### 3.3. Multivariable Logistic Regression Model

A multivariable logistic regression analysis showed that ever smokers had significantly higher odds of beta-blocker use compared with never smokers (OR = 3.53, 95% CI: 1.47–8.13, *p* = 0.0037). Male students had lower odds of beta-blocker use compared with females (OR = 0.40, 95% CI: 0.15–0.95, *p* = 0.048). No statistically significant association was observed between academic level and beta-blocker use (Table 3).

### 3.4. Prescription Status and Usage Patterns of Beta-Blockers Among Current Users

**Figure 2:**
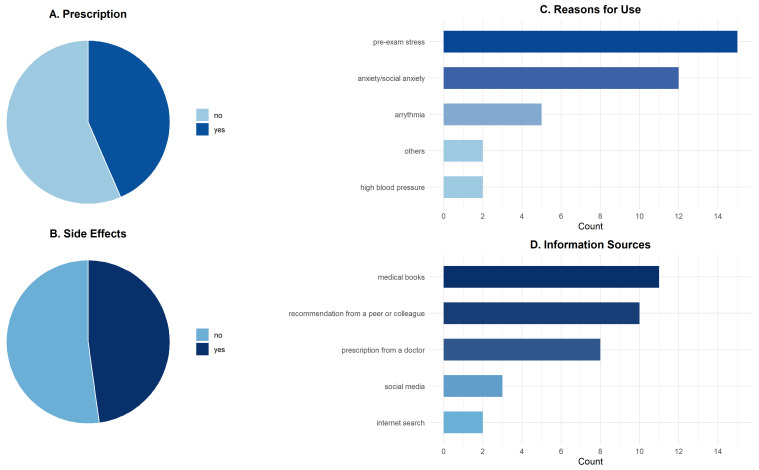
Beta-blocker prescription and its usage patterns among current users. (**A**) Prescription status. (**B**) Presence of side effects. (**C**) Reasons for beta-blocker use. (**D**) Sources of information about beta-blockers.

Among current beta-blocker users, the majority (56.5%) reported non-prescribed use, while 43.5% indicated that the medication was prescribed by a healthcare professional. Most commonly reported sources of gaining information about beta-blockers were medical books (47.8%) and peer or colleague recommendations (43.5%). The primary reasons for beta-blocker use were pre-exam stress (65.2%) and anxiety/social anxiety (52.2%). Propranolol was the most commonly used beta-blocker (78.3%). Most users reported using beta-blockers for more than six months (65.2%), and more than half used them only during examinations or public speaking situations (52.2%) (Figure 2, Table 4).

### 3.5. Perceived Effectiveness and Adverse Effects of Beta-Blockers Among Current Users

Most users found beta-blockers effective, with 52.2% rating them as very or extremely effective. Awareness of potential long-term risks was reported by 65.2% of users. Almost half of the users (47.8%) reported experiencing side effects, most commonly sleep problems, fatigue, nausea, and dizziness (Table 5).

### 3.6. Sleep Patterns and Sleep Quality After Starting Beta-Blocker Use Among Current Users

Several sleep-related disturbances were reported among beta-blocker users. While 47.8% reported no change in sleep duration, 30.4% reported an increase and 21.8% reported a decrease in sleep duration. The majority of current users (60.9%) reported no change in sleep quality, while 30.4% experienced a decline. Waking up more than once at night (73.9%), nightmares or disturbing dreams (69.6%), morning fatigue (65.2%), and difficulty waking up (52.2%) were also reported by current users. To a lesser extent, 39.1% of users reported sleep onset insomnia and 34.8% reported using sleep aids after they had started using beta-blockers, such as melatonin (Table 6).

## 4. Discussion

In our study, the overall prevalence of beta-blocker use was 4.9%, while only 3.3% used them currently. Most use was non-prescribed and situational, predominantly for exam-related stress and social anxiety. The main findings were sleep-related disturbances among users, including frequent night awakenings (73.9%), nightmares or disturbing dreams (69.6%), sleep-onset insomnia (39.1%), and self-reported poor sleep quality (30.4%). Despite these side effects, 52.2% of users perceived beta-blockers as very or extremely effective. Overall, these findings suggest that even intermittent use may be associated with clinically relevant adverse effects and highlight sleep quality as an underexplored issue.

### 4.1. Beta-Blockers and Sleep Disturbances

Different types of sleep disturbances were reported by beta-blocker users in this study, aligning with a previous study conducted on adults using beta-blockers, which reported that beta-blockers are an independent risk factor for sleep disturbances, with an estimated 29% increased risk [17]. A decline in sleep quality was reported by 30.4% of participants. A randomized trial comparing beta-blockers reported that 31.9% of patients receiving nebivolol were classified as poor sleepers, while a higher proportion was observed with metoprolol in the same study [21]. Furthermore, 39.1% of users experienced sleep-onset insomnia, which aligns with prior research associating beta-blocker use with insomnia [22,23]. These effects are biologically explained by β_1_-adrenergic blockade in the pineal gland, leading to suppression of melatonin secretion, a key hormone in circadian rhythm regulation [17,22,24].

However, these results should be cautiously interpreted, as sleep-related characteristics were assessed only descriptively among a small subgroup of current beta-blocker users. In addition, a lack of baseline data or comparison with non-users limits the ability to attribute the observed changes in sleep to beta-blocker use. Finally, important factors such as baseline anxiety, academic stress, depression, and pre-existing sleep disorders were not assessed, which may have contributed to the reported sleep-related symptoms.

Sleep duration showed variability, with 30.4% of users reporting increased duration and 21.8% reporting decreased duration. A large cohort study also demonstrated differences in sleep duration among beta-blocker users, with a median sleep duration of 8 h compared with 7 h in non-users, further supporting an association between beta-blocker exposure and altered sleep duration patterns [17]. This agrees with previous studies that beta-blockers may affect sleep differently across individuals, and that longer sleep duration does not necessarily indicate better sleep quality [25].

We further observed that 73.9% of beta-blocker users complained of waking more than once during the night. This is consistent with evidence associating lipophilic beta-blockers with increased night awakenings [26]. This finding can be attributed to melatonin suppression and disruption of circadian rhythm stability, as well as alterations in sleep architecture, including reduced slow-wave sleep and increased transitions between sleep stages [27].

Additionally, 69.6% of users reported nightmares or disturbing dreams, which aligns with previous studies associating beta-blockers with vivid dreaming [28,29,30]. This phenomenon may be explained by the disruption of REM sleep, the sleep stage most associated with dreaming, due to central nervous system effects of lipophilic beta-blockers [30].

Daytime effects were also prominent. Morning fatigue was reported by 65.2% of participants, whereas a Jordanian study among students using propranolol reported general fatigue in approximately half of the participants [31]. Although the two outcomes differ in timing and specificity, both suggest that fatigue-related symptoms are common in student users of beta-blockers. This may be explained by both central and peripheral effects of beta-blockers, which can be more pronounced during acute or intermittent use and may diminish with long-term adaptation [32].

Furthermore, 52.2% of users reported difficulty waking up in the morning. This finding appears underexplored in the literature, indicating that the full picture of the effects of beta-blocker use on sleep still needs further exploration.

### 4.2. Beta-Blocker Prescription Status and Patterns of Use

Propranolol was the most commonly used beta-blocker (78.3%) among users in our study. This aligns with existing literature in student populations where propranolol typically is the predominant, and often the only reported, beta-blocker used for performance anxiety [5,6,10]. This may be due to greater awareness and wider use of propranolol for performance anxiety, making it more commonly prescribed and recognized compared to other beta-blockers.

Another finding of this study was that 56.5% of participants reported using beta-blockers without a prescription, which aligns with previous studies showing a widespread culture of self-medication among Jordanian university students, including propranolol, where a previous study showed a similar percentage of 58.6% of students reporting unsupervised propranolol use [6,33], highlighting that self-medication remains a consistent and widespread behaviour in this population. This phenomenon is not only restricted to Jordan, as previous studies also demonstrated that physicians self-prescribe medications more than the general population, relying on their knowledge of medications and considering this a type of self-care behaviour [34].

Reasons for this use include medical students’ knowledge about medications in addition to the availability of certain beta-blockers, including propranolol, as drugs approved by the Jordan Food and Drug Administration (JFDA) in the over-the-counter (OTC) medication database, which may further facilitate non-prescribed access through community pharmacies [35]. This raises significant safety concerns, as unsupervised use may lead to inappropriate dosing, masking of underlying conditions, and increased risk of adverse effects [36].

One of the most common sources of information was peers (43.5%), paralleling earlier findings indicating that 44% of users learned about these medications through friends or peers [6]. This indicates that medication use is largely influenced by informal social networks rather than medical guidance and may contribute to misuse and inaccurate perceptions regarding the safety and indications of beta-blockers [37,38].

The primary reasons for use were pre-exam stress (65.2%) and social anxiety (52.2%). These findings are closely aligned with previous research among medical and dental students, which reported that the most common reasons for propranolol use were anxiety management (65.5%) and exam-related stress (60.3%) [6]. These motivations are pharmacologically explained as beta-blockers reduce peripheral adrenergic symptoms such as tachycardia and tremor, which are often interpreted as anxiety symptoms, thereby providing perceived relief without addressing the underlying psychological component [39].

Additionally, 52.2% of participants reported using beta-blockers only in specific situations, such as around exams or public speaking. This pattern reflects situational or performance-based use rather than therapeutic use and may contribute to increased psychological reliance on the medication [40]. The episodic nature of academic stress may make beta-blocker use more appealing to medical students because of its rapid effects compared to selective serotonin reuptake inhibitors (SSRI) and serotonin–norepinephrine reuptake inhibitors (SNRI). Although these medications are first-line treatments for anxiety disorders in many countries, the rapid effects of beta-blockers make them more appealing for medical students in Jordan [6,41,42,43]. However, most users (65.2%) had been using beta-blockers for more than six months, indicating a potential shift from occasional to prolonged use, which may increase the risk of long-term adverse effects.

### 4.3. Association Between Beta-Blocker Use and Demographic Factors

A significant association was observed between smoking status and beta-blocker use, with smokers being more likely to use beta-blockers. This may reflect higher baseline anxiety levels among smokers, leading to increased reliance on pharmacological coping strategies [44,45]. The prevalence of smoking in our sample (16.8%) was substantially lower than the 73.5% prevalence recently reported among Jordanian university students [46]. This difference may reflect the characteristics of our study sample, which consisted exclusively of medical students. The relationship between beta-blocker use and smoking needs to be investigated in future studies.

No significant associations were found with academic level or caffeine intake. However, multivariable analysis that male students had lower odds of beta-blocker use compared with females, suggesting that females were more likely to use beta-blockers. This finding may reflect gender-related differences in anxiety perception, help-seeking behaviour, or coping strategies [47].

### 4.4. Perceived Effectiveness and Adverse Effects

Despite the high prevalence of adverse effects, 52.2% of participants perceived beta-blockers as very or extremely effective. This perceived effectiveness is likely related to the reduction in physical symptoms of anxiety (e.g., heart rate, tremor), rather than an effect on psychological anxiety, leading users to overestimate the drug’s overall benefit [39].

Furthermore, 65.2% of users reported being aware of the potential long-term risks associated with beta-blocker use, consistent with previous findings showing that 74.1% of students were aware of the risks of unsupervised propranolol use [6]. However, this awareness did not prevent continued use, indicating a gap between knowledge and behaviour, which could be attributed to social desirability bias [48].

Almost half of the users in our study (47.8%) reported experiencing side effects, the most common being sleep problems, fatigue, nausea, dizziness, and cold extremities. This contrasts sharply with previous findings, where only 1.7% of students reported side effects [19]. This variation may be due to the broader range of side effects assessed in our study and potential differences in participant characteristics or recall of symptoms.

### 4.5. Limitations

This study has several limitations. First, the use of self-reported data may introduce recall and reporting bias. Second, the cross-sectional design limits causal inference. Additionally, sleep quality was assessed descriptively without a validated composite questionnaire such as the Pittsburgh Sleep Quality Index, which could have provided a more robust measurement. This questionnaire was not used because it limits answers to sleep habits during the prior month, whereas in our sample, we aimed to gain an understanding of the perceived effects of beta-blocker use on sleep regardless of the timeframe. The structured questionnaire used in this study was developed based on prior literature.

Additionally, sleep characteristics were assessed among current beta-blocker users without a non-user comparison group. Therefore, reported sleep disturbances cannot be attributed specifically to beta-blocker use, as medical students commonly experience sleep disturbances related to academic stress. Important potential confounding variables, including baseline anxiety, stress levels, depression, and pre-existing sleep disorders, were not assessed. Furthermore, the sample may not fully represent the population of all medical students, as female participants were overrepresented among the respondents, which may imply possible sampling bias. In addition, a substantial proportion of the participants were from the University of Jordan. Although this could be attributed to the higher number of medical students enrolled in this university compared to other universities in Jordan, it may limit the representativeness of the sample across all medical schools. Finally, the relatively small number of beta-blocker users, particularly current users, may limit the generalizability of the findings.

## 5. Conclusions

Beta-blocker use among medical students in Jordan is limited but present. Non-prescribed use is frequent, with propranolol being the most commonly used agent, primarily for anxiety and pre-exam stress. Use is significantly associated with smoking, with no link to gender, academic level, or caffeine intake. Sleep disturbances, including frequent nocturnal awakenings, nightmares, sleep onset insomnia, morning fatigue, and decreased sleep quality, are commonly reported among users. These findings highlight the importance of raising awareness about the potential adverse effects of unsupervised beta-blocker use on sleep and overall well-being and underscore the need for medical guidance when using these medications.

## Figures and Tables

**Table 1:** Sociodemographic characteristics of the study sample (*n* = 697).

Variable	Category	*n*	%
Age	<18	5	0.7
	18–21	470	67.4
	22–25	208	29.8
	>25	14	2.0
Gender	Female	484	69.4
	Male	213	30.6
Marital status	Single	687	98.6
	Married	10	1.4
Academic year	First	61	8.8
	Second	190	27.3
	Third	105	15.1
	Fourth	167	24.0
	Fifth	108	15.5
	Sixth	66	9.5
University	The University of Jordan	376	53.9
	Jordan University of Science and Technology	94	13.5
	Al-Balqa Applied University	71	10.2
	Yarmouk University	55	7.9
	Mutah university	53	7.6
	Hashemite University	48	6.9
Smoking status	Non-smoker	580	83.2
	Ex-smoker	17	2.4
	Smoker	100	14.3
Caffeine Consumption	Yes	598	85.8
	No	99	14.2
Chronic diseases	No	663	95.1
	Yes	34	4.9
Beta-blocker use	No	663	95.1
	Yes (current users)	23	3.3
	Yes (used in the past)	11	1.6

Percentages may not sum to 100% due to rounding.

**Table 2:** Association between beta-blocker use and demographic/lifestyle variables.

Variable	Categories	Non-Users		Users	%	df	X^2^	*p*-Value
		*n*	%	*n*				
Gender	Male	205	96.2%	8	3.8%	1	0.832	0.362
	Female	458	94.6%	26	5.4%			
Academic level	Preclinical years	343	96.3%	13	3.7%		2.359	0.125
	(first-third year)					1		
	Clinical years	320	93.8%	21	6.2%			
	(fourth-sixth)							
Smoking	Ever-smoker	106	90.6%	11	9.4%	1	6.201	0.013 *
	Never-smoker	557	96.0%	23	4.0%			
Caffeine consumption	No	93	93.9%	6	6.1%	1	0.348	0.555
	Yes	570	95.3%	28	4.7%			

* *p* < 0.05 statistically significant; Percentages may not sum to 100% due to rounding. Table 2 includes both current (*n* = 23) and past (*n* = 11) beta-blocker users (total *n* = 34).

**Table 3:** Multivariable logistic regression for factors associated with beta-blocker use.

Variable	OR	95% CI	*p*-Value
Ever smoker vs. Never smoker	3.53	1.47–8.13	0.0037 *
Male vs. Female	0.4	0.15–0.95	0.048 *
Preclinical vs. Clinical years	0.63	0.30–1.28	0.211

* *p* < 0.05 statistically significant. Table 3 includes both current (*n* = 23) and past (*n* = 11) beta-blocker users (total *n* = 34). Reference categories: Never smoker (smoking status), Female (sex), Clinical years (academic level).

**Table 4:** Beta-blocker prescription and usage patterns among current users (*n* = 23).

Variable	Category	*n*	%
Prescribed by a healthcare professional	Yes	10	43.5
	No	13	56.5
First learned about beta-blockers *	Prescription from a doctor	8	34.8
	Recommendation from a peer/colleague	10	43.5
	Social media	3	13.0
	Medical books	11	47.8
	Internet search	2	8.7
Reason for using beta-blockers *	Anxiety/social anxiety	12	52.2
	Pre-exam stress	15	65.2
	Arrhythmia	5	21.7
	High blood pressure	2	8.7
	Others	2	8.7
Type of beta-blockers used	Propranolol	18	78.3
	Atenolol	3	13.0
	Timolol	1	4.3
	Metoprolol	1	4.3
When did you start using beta-blockers	<1 month	3	13.0
	1–3 months	2	8.7
	3–6 months	3	13.0
	>6 months	15	65.2
How often do you use beta-blockers	Daily	8	34.8
	Several times a week	1	4.3
	Less than once a week	2	8.7
	Only on exam/public speaking	12	52.2

* Multiple responses were allowed; percentages may not sum to 100% due to rounding.

**Table 5:** Perceived effectiveness and side effects of beta-blockers among current users (*n* = 23).

Variable	Category	*n*	%
Effectiveness of beta-blockers	Not effective at all	0	0
	Effective	6	26.1
	Somewhat effective	5	21.7
	Very effective	7	30.4
	Extremely effective	5	21.7
Awareness of long-term risks	Yes	15	65.2
	No	8	34.8
Experienced side effects	Yes	11	47.8
	No	12	52.2
Type of side effects among those who experienced them *	Nausea	6	54.5
	Bradycardia	3	27.3
	Cold extremities	3	27.3
	Sleep problems	7	63.6
	Fatigue	6	54.5
	Weight gain	2	18.2
	Dizziness	6	54.5

* Multiple responses were allowed; Percentages may not sum to 100% due to rounding.

**Table 6:** Sleep patterns and sleep quality after starting beta-blocker use among current users (*n* = 23).

Variable	Category	*n*	%	95% CI
Change in sleep duration	Increased	7	30.4	16.0–51.0
	Decreased	5	21.7	10.0–42.0
	Did not change	11	47.8	29.0–67.0
Change in sleep quality	Improved	2	8.7	2.0–27.0
	Declined	7	30.4	16.0–51.0
	Did not change	14	60.9	41.0–78.0
Time to fall asleep	<15 min	4	17.4	7.0–37.0
	15–30 min	9	39.1	22.0–59.0
	30–60 min	4	17.4	7.0–37.0
	>60 min	6	26.1	13.0–47.0
Waking up more than once at night	Yes	17	73.9	54.0–87.0
	No	6	26.1	
Nightmares or disturbing dreams	Yes	16	69.6	49.0–84.0
	No	7	30.4	
Sleep-onset insomnia	Yes	9	39.1	22.0–59.0
	No	14	60.9	
Difficulty waking up	Yes	12	52.2	33.0–71.0
	No	11	47.8	
Morning sleepiness/fatigue	Yes	15	65.2	45.0–81.0
	No	8	34.8	
Daytime sleep increase	Yes	6	26.1	13.0–47.0
	No	17	73.9	
Morning energy level	Increased	1	4.3	1.0–21.0
	Decreased	7	30.4	16.0–51.0
	Did not change	15	65.2	45.0–81.0
Morning mental motivation	Increased	8	34.8	19.0–55.0
	Decreased	2	8.7	2.0–27.0
	Did not change	13	56.5	37.0–74.0
Use of sleep aids (like melatonin)	Yes	8	34.8	19.0–55.0
	No	15	65.2	

Percentages may not sum to 100% due to rounding.

## Data Availability

The data presented in this study are available on request from the corresponding author due to institutional and ethical restrictions.

## Supplementary Materials

The following supporting information can be downloaded at https://www.mdpi.com/article/10.3390/healthcare14152257/s1, Bilingual Survey Questions.
