# Supplementary material for: Beta-Blocker Use and Self-Reported Sleep Changes Among Medical Students in Jordan: A Cross-Sectional Study
Source: Healthcare (Basel). 2026 Jul 24;14(15):2257. doi: 10.3390/healthcare14152257 (PMC13465002; doi:10.3390/healthcare14152257)
Supplement: Supplementary file 1 [file healthcare-14-02257-s001.zip › healthcare-4291192-supplementary.pdf]

ملحق أسئلة الاستبيان (Bilingual Survey Questions)

We are a group of medical students from Jordan conducting a study that aims to evaluate the effect of beta-blockers use on sleep quality among medical students. You are invited to participate in this study by completing a short questionnaire that includes questions about this medication use, sleep quality, and some general information. Your participation is entirely voluntary, and you have the right to refuse participation or withdraw at any time without any consequences. No personal identifying information will be collected and all responses will be kept strictly confidential and used for scientific research purposes only.<br><br>Do you agree to have your responses used for research purposes while ensuring complete confidentiality?	نحن مجموعة من طلبة الطب في الأردن نقوم بإجراء دراسة تهدف إلى تقييم تأثير استخدام حاصرات بيتا (Beta Blockers) على جودة النوم لدى طلبة الطب. ندعوك للمشاركة في هذه الدراسة من خلال تعبئة استبيان قصير يتضمن أسئلة حول استخدام الأدوية وجودة النوم وبعض المعلومات العامة. مشاركتك في هذه الدراسة طوعية تمامًا، ويحق لك رفض المشاركة أو الانسحاب في أي وقت دون أي تبعات. لن يتم جمع أي مع تعريفية شخصية، وستعامل جميع الإجابات بسرية تامة وتستخدم لأغراض البحث العلمي فقط.<br><br>هل توافق على ان تستخدم اجوبتك لدواعي البحث مع الحفاظ على السرية التامة؟
• Yes<br>• No	• نعم<br>• لا

Q1: Age	س ١: العمر
• Less than 18 years<br>• 18–21 years<br>• 22–25 years<br>• More than 25 years	• أقل من 18<br>• 18-22<br>• 22-25<br>• أكبر من 25

Q2 Sex	س.2 الجنس:
• Male<br>• Female	• أنثى<br>• ذكر

Q3 Marital status	س3. الحالة الاجتماعية:
• Single<br>• Married<br>• Divorced	• أعزب / عزباء<br>• متزوج / متزوجة<br>• مطلق / مطلقة

Q 4 Academic year	**س4. السنة الدراسية:**
• Year 1<br>• Year 2<br>• Year 3<br>• Year 4<br>• Year 5<br>• Year 6	• أولى<br>• ثانية<br>• ثالثة<br>• رابعة<br>• خامسة<br>• سادسة

Q 5 University	س5. الجامعة:
• University of Jordan<br>• Hashemite University<br>• Jordan University of Science and Technology<br>• Mutah University<br>• Al-Balqa Applied University<br>• Yarmouk University	• الجامعة الأردنية<br>• الجامعة الهاشمية<br>• جامعة العلوم والتكنولوجيا<br>• جامعة مؤ□ة<br>• جامعة البلقاء التطبيقية<br>• جامعة اليرموك

Q 6 Are you a smoker?	س6. هل أنت مدخن؟
• Yes<br>• No<br>• Ex-smoker	• نعم<br>• لا<br>• مدخن سابق

Q 7 Do you consume caffeinated products (Coffee-Tea-Energy Drinks)?	س7. هل□ستهلك منتجات الكافيين (القهوة، الشاي، مشروبات الطاقة)؟

• Yes<br>• No	• نعم<br>• لا

Q 8 Do you suffer from any chronic diseases?	س8. هل□عاني من أي أمراض مزمنة؟
• Yes<br>• No	• نعم<br>• لا

Q 9 If yes, mention your chronic diseases	س9. إذا كانت الإجابة نعم، يرجى ذكر الأمراض المزمنة التي□عاني منها:
……………………………………..	……………………………………..

Q 10 Do you use or have ever used beta blockers?	س10. هل□ستخدم أو سبق لك أن استخدمت حاصرات بيتا؟
• Yes<br>• Used them previously<br>• No	• نعم<br>• استخدمتها سابقا<br>• لا

أسئلة لمستخدمي حاصرات بيتا (حاليا او سابقا)

Questions for beta-blocker users (current and past users)

Q 11 How did you first learn about beta blockers?<br>(You can choose multiple answers)	س11. كيف□عرفت لأول مرة على حاصرات بيتا؟ (يمكن اختيار أكثر من جواب)
• Direct prescription from a doctor<br>• Recommendation from a friend / colleague<br>• Internet search<br>• Social media<br>• Medical school curriculum	• وصف□بي مباشر من الطبيب<br>• □وصية من صديق / زميل<br>• بحث عبر الإنترنت<br>• موقع□واصل اجتماعي<br>• مناهج كلية الطب

Q 12 Why are you using, or why have you used, beta blockers?<br>(You can choose multiple answers)	س12. ما سبب استخدامك لحاصرات بيتا؟<br>(يمكن اختيار أكثر من جواب)
• Hypertension (High blood pressure)<br>• Arrhythmia (Heart rhythm disorder)<br>• Anxiety or social phobia<br>• Pre-examination stress<br>• Others	• ار□فاع ضغط الدم<br>• اضطراب نظم القلب<br>• القلق أو الرهاب الاجتماعي<br>• التو□ر ما قبل الامتحان<br>• سبب آخر

Q 13 Have you been prescribed a beta blocker by a healthcare professional?	س13. هل□م وصف حاصر بيتا لك من قبل مختص صحي؟
• Yes<br>• No	• نعم<br>• لا

Q 14 What is the name of the beta blocker you are using?	س14. ما اسم حاصر بيتا الذي□ستخدمه؟ (يرجى كتابة الاسم):
....	....

Q15. When did you start using beta-blockers?	س15. منذ متى بدأت باستخدام حاصرات بيتا؟
• Less than a month<br>• 1 to 3 months<br>• 3 to 6 months<br>• More than 6 months	• أقل من شهر<br>• من 1 إلى 3 أشهر<br>• من 3 إلى 6 أشهر<br>• أكثر من 6 أشهر

Q 16 How often do you use beta blockers?	س16. ما مدى□كرار استخدامك لحاصرات بيتا؟

• Daily<br>• Several times a week<br>• Occasionally (less than once a week)<br>• Only on exam days or public speaking (as needed)	• يومياً<br>• عدة مرات في الأسبوع<br>• أحياناً (أقل من مرة في الأسبوع)<br>• فقط في أيام الاختبارات أو التحدث أمام الجمهور (حسب الحاجة)
Q 17 What time of day do you usually take beta blockers?	س17. في أي وقت من اليوم□تناول حاصرات بيتا عادة؟
• In the morning<br>• In the evening<br>• As needed only<br>• Random (no specific time)	• في الصباح<br>• في المساء<br>• حسب الحاجة فقط<br>• عشوائي (لا وقت محدد)
Q 18 How effective are beta blockers in controlling your symptoms?	س18. ما مدى فعالية حاصر بيتا في التحكم بأعراضك؟
• Extremely effective<br>• Very effective<br>• Effective<br>• Somewhat effective<br>• Not effective at all	• فعّال للغاية<br>• فعّال جداً<br>• فعّال<br>• فعّال إلى حد ما<br>• غير فعّال إ□لاقا
Q 19 If you use beta blockers during times of stress, how long before the stressful event do you usually take the beta blocker?	س19. في حال استخدام الحاصرات في أوقات التو□ر، كم من الوقت قبل الحدث المسبب للتو□ر□تناول حاصر بيتا عادةً؟
• Less than 30 minutes<br>• 30 to 60 minutes<br>• 1 to 2 hours<br>• More than 2 hours<br>• I don't use them in stressful situations	• أقل من 30 دقيقة<br>• من 30 إلى 60 دقيقة<br>• من ساعة إلى ساعتين<br>• أكثر من ساعتين<br>• لا أستخدمها في حالات التو□ر
Q 20 Are you aware of the potential risks of long-term use of beta blockers?	س20. هل أنت على علم بالمخا□ر المحتملة للاستخدام □ويل المدى لحاصر بيتا؟

• Yes<br>• No	• نعم<br>• لا
Q 21 Would you recommend beta blockers to others?	س21. هل□وصي باستخدام حاصر بيتا للآخرين؟
• Yes<br>• No	• نعم<br>• لا

Q 22 Have you ever experienced any side effects from beta blockers?	س22. هل سبق أن عانيت من أي آثار جانبية لحاصر بيتا؟
• Yes<br>• No	• نعم<br>• لا

Q 23 What side effects did you experience?<br>(You can choose multiple answers)	س23. ما هي الآثار الجانبية التي عانيت منها؟ (يمكن اختيار أكثر من إجابة)
•<br>• Dizziness<br>• Nausea<br>• Cold hands or extremities<br>• Fatigue<br>• Slow heart rate (Bradycardia)<br>• Sleep problems (insomnia, interrupted sleep, nightmares)<br>• Weight gain	• دوار<br>• غثيان<br>• برودة اليدين أو الأ□راف<br>• إرهاق<br>• بطء ضربات القلب (بطء القلب)<br>• مشاكل في النوم (مثل أرق، نوم متقطع، كوابيس)<br>• زيادة الوزن

Q 24 Has your daily stress level changed after taking beta blockers?	س24. هل تغير مستوى الضغط النفسي اليومي الذي تشعر به بعد تناولك حاصرات البيتا؟
• Decreased slightly<br>• Decreased moderately<br>• Decreased significantly<br>• Increased slightly<br>• Increased moderately	• أقل بشكل بسيط<br>• قل بشكل متوسط<br>• قل بشكل كبير<br>• ازداد بشكل بسيط<br>• ازداد بشكل متوسط

• Increased significantly<br>• Has not changed	• ازداد بشكل كبير<br>• لم يتغير

Q 25 Have you noticed a change in your average number of hours of sleep after starting beta blockers?	س25. هل لاحظت□غيرًا في متوسط عدد ساعات نومك بعد بدء استخدام حاصرات بيتا؟
• Yes, my average number of sleep hours increased<br>• Yes, my average number of sleep hours decreased<br>• No, I haven't noticed any change	• نعم، ازداد متوسط عدد ساعات نومي<br>• نعم، قل متوسط عدد ساعات نومي<br>• لا، لم ألاحظ أي□غيير

Q 26 Have you noticed a change in your sleep quality after starting beta blockers?	س26. هل لاحظت□غيرًا في جودة نومك بعد بدء استخدام حاصرات بيتا؟
• Yes, my sleep quality improved<br>• Yes, my sleep quality deteriorated<br>• No, I haven't noticed any change	• نعم□حسنت جودة نومي<br>• نعم□دهورت جودة نومي<br>• لا، لم ألاحظ أي□غيير

• Q 27 How long does it usually take to start sleeping after using beta blockers?	س27. كم الوقت الذي□حتاجه عادةً لتبدأ النوم بعد استخدام حاصرات بيتا؟
• Less than 15 minutes<br>• 15 to 30 minutes<br>• 30 to 60 minutes<br>• More than 60 minutes	• أقل من 15 دقيقة<br>• من 15 إلى 30 دقيقة<br>• من 30 إلى 60 دقيقة<br>• أكثر من 60 دقيقة

Q 28 Has your sleep become interrupted or unstable after using beta blockers?	س28. هل أصبح نومك متقطعًا أو غير مستقر بعد استخدام حاصرات بيتا؟
• Yes<br>• No	• نعم<br>• لا

Q 29 Have you been waking up frequently during the night since starting beta blockers?	س29. هل□عاني من الاستيقاظ المتكرر أثناء الليل منذ بدء استخدام حاصرات بيتا؟
• Yes<br>• No	• نعم<br>• لا

Q 30 Have you started experiencing nightmares or disturbing dreams after using beta blockers?	س30. هل بدأت□عاني من كوابيس أو أحلام مزعجة بعد استخدام حاصرات بيتا؟
• Yes<br>• No	• نعم<br>• لا

Q 31 Do you have difficulty falling asleep (sleep-onset insomnia) after using beta blockers?	س31. هل□واجه صعوبة في البدء بالنوم (أرق في بداية الليل) بعد استخدام حاصرات بيتا؟
• Yes<br>• No	• نعم<br>• لا

Q 32 Have you had difficulty waking up in the morning since starting beta blockers?	س32. هل أصبحت□جد صعوبة في الاستيقاظ صباحًا منذ بدء استخدام حاصرات بيتا؟
• Yes<br>• No	• نعم<br>• لا

Q 33 Have you felt sleepy or tired immediately after waking up, even without doing any exercise, since starting beta blockers?	س33. هل□شعر بالنعاس أو الإرهاق فور الاستيقاظ، حتى دون القيام بأي مجهود؟
• Yes<br>• No	• نعم<br>• لا

Q 34 Have you noticed an increase in daytime sleep (such as napping or frequent sleep-in) after using beta blockers?	س34. هل لاحظت زيادة في فترات النوم خلال النهار (مثل القيلولة أو النوم المتكرر) بعد استخدام حاصرات بيتا؟
• Yes<br>• No	• نعم<br>• لا

Q 35 Have you noticed a difference in your energy level during the first few hours after waking up after using beta blockers?	س35. هل لاحظت فرقًا في مستوى الطاقة البدنية خلال أول ساعات ما بعد النوم بعد استخدام حاصرات بيتا؟
• Increased<br>• Decreased<br>• I haven't noticed a difference	• ازداد<br>• انخفض<br>• لم ألاحظ فرقا

Q 36 Have you noticed a difference in your level of mental motivation during the first few hours after waking up after using beta blockers?	س36. هل لاحظت فرقًا في مستوى الحافز النفسي خلال أول ساعات ما بعد الاستيقاظ بعد استخدام حاصرات بيتا؟
• Increased<br>• Decreased<br>• I haven't noticed a difference	• ازداد<br>• انخفض<br>• لم ألاحظ فرقاً

Q 37 Did you have to take any sleep aids (such as melatonin) while taking beta blockers?	س37. هل اضطررت إلى تناول أي أدوية مساعدة للنوم (مثل الميلاتونين) أثناء تناول حاصرات بيتا؟
• Yes<br>• No	• نعم<br>• لا
